# Identification of several hub-genes associated with periodontitis using integrated microarray analysis

**DOI:** 10.3892/mmr.2014.3031

**Published:** 2014-12-02

**Authors:** XINXING GUO, YILING WANG, CHUNLING WANG, JING CHEN

**Affiliations:** 1 Department of Orthodontics, School of Stomatology, Shandong University, Jinan, Shandong 250012, P.R. China; 2 Department of Orthodontics, Jinan Stomatological Hospital, Jinan, Shandong 250001, P.R. China; 3 Department of Sterilization and Supply Center, School of Stomatology, Shandong University, Jinan, Shandong 250012, P.R. China

**Keywords:** periodontitis, differentially expressed genes, bioinformatics

## Abstract

The aim of the present study was to identify differentially expressed genes and biological processes associated with periodontitis. In this study, the most significant 200 differentially expressed genes associated with periodontitis were identified using integrated analysis of multiple microarray data in combination with screening for genome-wide relative significance and genome-wide global significance. Gene Ontology (GO) enrichment analysis and pathway analysis were performed using the GO website and Kyoto Encyclopedia of Genes and Genomes (KEGG). A protein-protein interaction (PPI) network was constructed based on the Search Tool for the Retrieval of Interacting Genes/Proteins. The top 200 differentially expressed genes were found to be highly associated with periodontitis. GO enrichment analyses revealed that the identified genes were significantly enriched in terms of response to organic substance, response to wounding and cell migration. The most common term of the KEGG pathway was cytokine-cytokine receptor interaction. PPI network analysis indicated that interleukin (IL)8, IL1β, vascular endothelial growth factor A, intercellular adhesion molecule 1, PTGS2 and CXCL10 were hub genes, which formed numerous interactions with several genes. In conclusion, the present study identified numerous genes that were differentially expressed in periodontitis, as well as determined the biological pathways and PPI network associated with those genes.

## Introduction

Periodontitis is a common chronic infection of the supporting tissues of teeth, which has been epidemiologically associated with cardiovascular diseases (1). Previous studies have indicated that periodontitis is more than a localized oral infection and it may have marked effects on systemic health (2,3).

Numerous clinical and scientific studies have provided evidence that the risk of adult periodontitis has a genetic component (4–6). Genetic factors are important for assessing the susceptibility of patients to periodontitis and the rate of its progression, as demonstrated by studies in humans and animals, which have indicated that genetic factors influence general inflammatory and immune responses (7,8). Individuals have differential responses to common environmental stimuli, which is dependent on the genetic profile of an individual (9). Different forms of genes (allelic variants) may induce variations in tissue structure (innate immunity), antibody responses (adaptive immunity) and inflammatory mediators (non-specific inflammation) (10). In order to elucidate the potential effect of a patient’s genetic profile on periodontitis, the contribution of different genes to the pathogenesis of periodontitis must be understood. Therefore, identification of the most important genes that contribute to the development of this disease is essential.

Analyses of differential gene expression, performed using high-throughput experimental methods, such as microarray analysis, have been used in an increasing number of studies in recent years (11,12). At present, a vast quantity of microarray data have been produced and deposited in publically-available data repositories, including Gene Expression Omnibus (GEO; http://www.ncbi.nlm.nih.gov/geo/) (13) and Array Express Archive (http://www.ebi.ac.uk/arrayexpress/) (14). These repositories allow researchers to advance the identification of genetic and diagnostic signatures using data integration and bioinformatics analysis. This may therefore provide insights into the biological mechanisms underlying the development of periodontitis. Demmer *et al* (15) revealed that 5,295 genes were upregulated and 7,449 genes were downregulated in periodontitis using transcriptome analysis. Gene ontology (GO) analysis identified groups of differently expressed genes (DEGs) in periodontitis, including those involved in apoptosis, antimicrobial humoral responses and antigen presentation. Becker *et al* (16) also presented a disease-associated mRNA profile, which displayed the potential underlying mechanisms of periodontitis. GO analysis revealed that the regulation of transcripts associated with bacterial response systems were dominant in periodontitis tissues. However, microarray-based biomarkers have had poor translation into clinical practice; furthermore, the results were non-conformal among studies due to small sample sizes.

A robust genetic marker signature may be beneficial to the diagnosis and targeted treatment of periodontitis in clinical practice. In the present study, in order to identify a more credible gene biomarker signature for periodontitis, an integrated analysis of microarray data was performed using a novel model that screened for genome-wide relative significance (GWRS) and genome-wide global significance (GWGS) (17). The present study also aimed to build a more precise target network for periodontitis using the selected biomarkers and then further investigate the identified DEGs through functional enrichment analysis, pathway enrichment analysis and protein-protein interaction (PPI) networks.

## Materials and methods

### Identification of gene expression datasets

**Figure 1:**
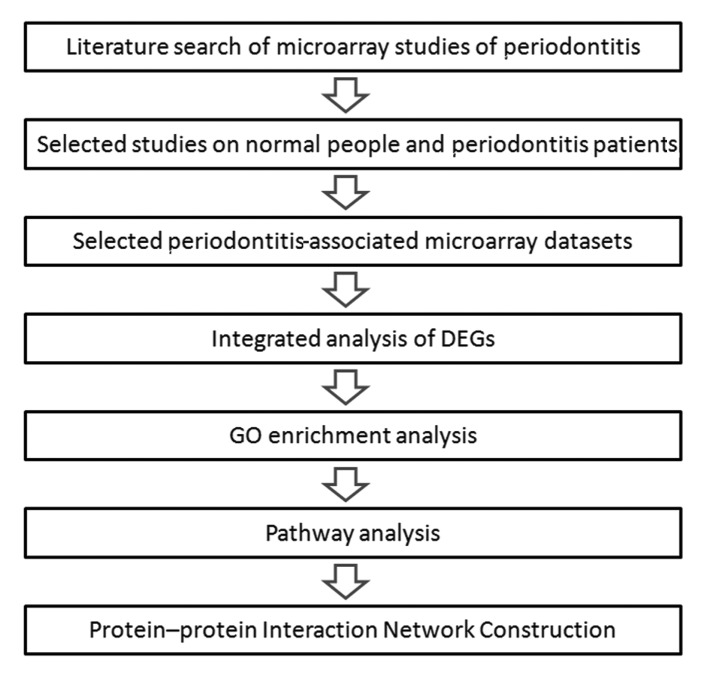
Experimental protocol of the present study. DEG, differentially expressed genes; GO, gene ontology.

In the present study, DEGs were identified between normal healthy volunteers and periodontitis patients. Between January 2008 and January 2014, three microarray datasets were extracted from the National Center for Biotechnology Information GEO database: GEO access nos. GSE10334 (15), GSE27993 (18) and GSE33774 (16). The experimental protocol for the present study is shown in Fig. 1. The following information was extracted from each identified study: GEO accession number, sample type, platform, number of cases and controls, and gene expression data. Animal studies and studies in which microarray data were uncertain were excluded.

### Integrated analysis of DEGs from multiple microarray data

Data pre-processing was performed using the Bioconductor XPS package v1.24.1 (www.bioconductor.org/packages/release/bioc/html/xps.html) (19), which was based on the data analysis framework ROOT v5 (http://root.cern.ch). Probes were excluded when they did not match a corresponding gene symbol; when a probe-set was mapped to multiple genes, all of these genes were given the expression of the probe-set. Maxim-based methods were used to deal with the situations where multiple probe-sets were associated with a gene. Microarry Suite 5 v7.3–35 (Affymetrix, Inc., Santa Clara, CA, USA) was used to compute expression levels. For each dataset, P<0.05 and a false discovery rate value<0.05 were set as the parameters for DEGs.

### Integrated analysis of DEGs identified in the three microarray datasets using GWRS and GWGS

The gene signatures were identified using a novel model which measured the GWRS and GWGS of gene expression. The GWRS of a gene was measured using its ranking position on a genome-wide scale based on the differential expression measured in each individual microarray dataset The number of datasets was denoted by *n*, the number of unique genes across *n* datasets was denoted by *m*; GWRS of the *i*-th gene in the *j*-th dataset was measured by: ${\text{s}}_{\text{ij}}=-2(\text{log}\frac{{\text{r}}_{\text{ij}}}{\text{m}})$, where r_ij_, *i*=1-*m*, *j*=1-*n*, was the rank number of the *i*-th gene in the *j*-th study. GWGS was applied to synthetically analyze multiple microarray studies. The GWGS of a gene was estimated based on its corresponding GWRS across *n* datasets. GWGS of a gene was calculated by: s_i_^r^ = ∑_j=1_^n^ ω_j_s_ij_, where w_j_ represented the relative weight of the *j*-th dataset. These calculations were performed as previously described (17). A gene with a large GWGS value was considered to be globally significant across multiple independent studies.

In the present study, the degree of differential gene expression was measured by a fold-change based algorithm, which was found to be more suited to measure the significance of differential expression compared with that of other statistical tests which give P-values and the significance analysis of microarray (SAM). The fold-change-based differential expression analysis was conducted using the Linear Models for Microarray Data (LIMMA) package (http://bioinf.wehi.edu.au/limma/) (20). A rank number was assigned to each gene according to its degree of differential expression (in descending order from 1 to *m*) and equal weight was assigned to each data. The smallest number of genes that may achieve a suitable level of classification performance was 200; therefore, the top 200 genes were selected for further analysis.

### Functional and pathway enrichment analysis

In order to further investigate the functions of the DEGs, GO-categories were organized based on the GO database (http://www.geneontology.org/). The enrichment analyses required >5 genes to be present and P<0.01 for a category to be considered significant. In order to further investigate the signaling pathway of the DEGs, pathway analysis based on the Kyoto Encyclopedia of Genes and Genomes (KEGG) database (www.genome.jp/kegg/) was performed. The two analyses were performed using the Database for Annotation, Visualization and Integrated Discovery (DAVID; http://david.abcc.ncifcrf.gov/home.jsp) (21). The significant categories were identified by expression analysis systematic exprorer (EASE) scores, a modified Fisher Exact P-value. The threshold for significance for a category was P<0.05, with >2 genes for the corresponding term.

### PPI network construction

The PPI network is a useful research tool for investigating the cellular networks of protein interactions. A PPI network was constructed for the top 200 DEGs. Interaction data were obtained from the Search Tool for the Retrieval of Interacting Genes/Proteins (STRING; http://string.embl.de/). DEGs were then imported into the interaction network and interactions were screened with both end nodes having DEGs. All nodes with the degree ≥1 were reserved. The networks were constructed using Cytoscape 3.1.0 (http://www.cytoscape.org/).

## Results

### Integrated analysis of DEGs in multiple studies

**Figure 2:**
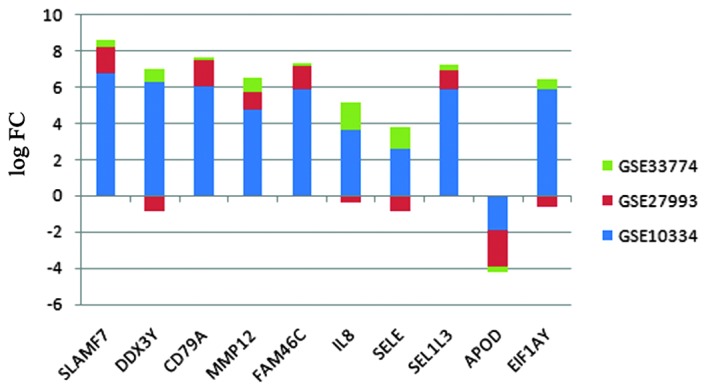
Expression pattern of the top 10 ranked differentially expressed genes identified by the integrated analysis of three microarray databases. FC, fold change.

Information was extracted from the microarray datasets GSE10334, GSE27993 and GSE33774 for 9,925, 8,322 and 6609 genes, respectively, following the exclusion of animal and uncertain data as well as duplicated genes. Following intersection of the microarray datasets, 2,754 common genes were identified. The GWGS values of genes were then applied to the integrated independent microarray data. Genes with large GWGS values were considered to be globally significant across multiple independent studies as genes with larger fold-changes were ranked highly for individual microarray. Following the intersection of the three microarray datasets, the top 200 DEGs were identified (Table I). The expression pattern of the top 10 genes is presented in Fig. 2.

### Functional and pathway enrichment analysis

In order to further investigate the functions of the identified top 200 genes, pathway and GO analysis were performed. A total of 135 terms were retrieved from the DAVID database.

Pathway analysis revealed genes that were significantly enriched in six terms; the most common three terms were those involved in cytokine-cytokine receptor interactions (P=2.6×10^−6^), cell adhesion molecules (P=1.1×10^−2^) and complement and coagulation cascades (P=1.6×10^−2^) (Table II).

GO analysis revealed that the identified genes were predominantly involved in biological processes of immune response (P=6.6×10^−9^), response to organic substances (P=1.1×10^−5^) and response to wounding (P=1.3×10^−5^). The top 10 GO terms are shown in Table III.

### Structure interaction network of the DEGs

**Figure 3:**
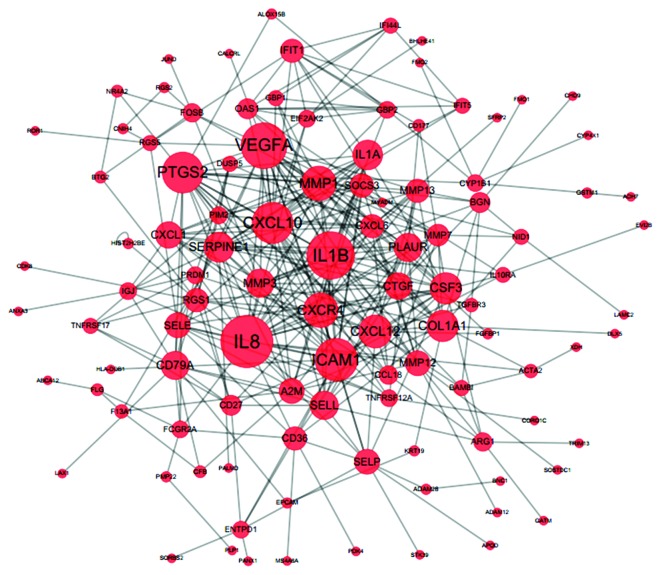
Protein-protein interaction networks of the top 200 differentially expressed genes identified by the integrated analysis of three microarray databases. Each biological relationship (an edge) between two genes (nodes) is supported by at least one reference from the literature or information stored in the Search Tool for the Retrieval of Interacting Genes/Proteins Base. The degree for each gene is represented by the size of the gene node.

Based on the STRING database, the interaction networks of the identified DEGs were constructed, which consisted of 332 edges (biological associations) and 106 nodes (genes that form associations) (Fig. 3). Genes with a high degree of association included interleukin (IL)8, IL1β, vascular endothelial growth factor A (VEGFA), intercellular adhesion molecule 1 (ICAM1), PTGS2 and CXCL10. Genes with degrees >10 in the PPI network are show in Table IV.

## Discussion

GWGS has been demonstrated to be a reliable method for comparing microarray data from different sources and studies (17). Based on this strategy, the analysis of the present study focused on significantly DEGs in order to reveal the transcriptional responses of each periodontitis sample. The results of this analysis indicated that these genes were enriched in cytokine-cytokine receptor interactions, cell adhesion molecules as well as complement and coagulation cascades.

Hub nodes are genes highly connected with other genes and have been identified to have important roles in numerous networks. In addition, highly connected hub genes were proposed to have an important role in biological development (22). Hub nodes have more complex interactions compared with those of other genes, which indicates that they have pivotal roles in the underlying mechanisms of disease. Identification of the hub genes involved in periodontitis may lead to the development of effective diagnostic and therapeutic approaches for periodontitis. In the present study, DEGs, which were algorithmically predicted to be biomarkers for periodontitis using GWGS, were found in high levels within patients with periodontitis. In addition, certain identified biomarkers, including IL8, IL1β, ICAM1 and VEGFA, were hub genes, therefore suggesting that they may be useful diagnostic markers for periodontitis.

In the present study, the IL8 gene had a degree of 28, which was the highest degree in the PPI network. IL8 is a member of the family of chemokines that mediates the activation and migration of neutrophils from peripheral blood into tissues (23). IL8 expression may be induced by IL18 in natural killer cells, which was reported to be inhibited by tumor necrosis factor binding protein (24). Kimata *et al* (25) demonstrated that IL8 selectively inhibited immunoglobulin (Ig)E and IgG4, which were induced by IL4; however, IL8 exerted no effects on the production of IgM, IgG1, IgA, IgG2 and IgG3. A previous study suggested that chronic stress was associated with increased IL8 levels (26). This was consistent with another study, which reported that individuals with high levels of trait anxiety were more prone to periodontal disease (27).

IL1β is also a potent pro-inflammatory cytokine, which is upregulated by radiation and is involved in the regulation of other inflammation-associated molecules (28). In cultured human umbilical vein endothelial cells, IL1β activated VCAM-1 gene expression (29). Several previous studies have demonstrated strong associations between the high frequency of genetic polymorphism in IL1β and the development of severe chronic periodontitis (30–32). Gursoy *et al* (33)revealed that IL1β was the only biomarker associated with periodontitis among the salivary cytokines and enzymes tested. In addition, IL1β polymorphisms were reported to be associated with an increased risk of cancer (34,35).

In the present study, the VEGFA gene was also found to have a high degree of 24 in the PPI network. VEGFA is the predominant mediator of angiogenesis in the VEGF family (36) and is essential for chondrocyte survival during bone development (37). A previous study demonstrated that VEGFA stimulated lymphangiogenesis and hemangiogenesis in inflammatory neovascularization via macrophage recruitment (38). In a study by Kasprzak *et al* (39) VEGF was overexpressed in patients with chronic periodontitis, suggesting its significance in protracting the inflammatory process or periodic exacerbations of the process and the destruction of the periodeontium. In addition, Tian *et al* (40) reported that the genotypes of VEGF and its protein production were associated with chronic periodontitis in a Chinese population. Furthermore, VEGFs were also found to have an important role in neurodegeneration (41).

ICAM1 was reported to have a role in the onset and manifestation of the inflammatory responses. ICAM1 was identified as a co-stimulatory ligand, which bound to lymphocyte function-associated antigen-1 (42); in addition, ICAM1 was reported to contribute to the adhesion of T lymphocytes to chondrocytes (43). A previous study demonstrated that ICAM1 was highly expressed in infected gingival cells as well as in tissues from periodontitis patients compared with those from healthy controls (44). Nedbal *et al* (45) suggested that the local topical applications of ICAM1-directed antisense oligonucleotides may be used as an effective therapeutic tool against the inflammatory processes of the human gingival. Furthermore, ICAM1 expression has been reported to be associated with other diseases including Alzheimer’s Disease (46) and cancer (47).

In conclusion, the present study identified several DEGs associated with periodontitis, as well as the functions and signaling pathways in which these genes were involved. Comprehensive network analyses of the dysregulated gene expression in periodontitis identified a series of hub genes that had high degrees of PPI in this network. This PPI network was therefore proposed to reflect the interaction of genes in the periodontitis-specific micro-environment. In addition, the hub genes identified may be potential useful diagnostic markers and novel therapeutic targets for periodontitis.

## Figures and Tables

**Table I:** Top 200 differentially expressed genes identified by the integrated analysis of three microarray datasets.

No.	Gene
1	SLAMF7
2	DDX3Y
3	CD79A
4	MMP12
5	FAM46C
6	IL8
7	SELE
8	SEL1L3
9	APOD
10	EIF1AY
11	SLC7A11
12	CD27
13	MMP7
14	ADAM28
15	IL1β
16	LAX1
17	IGLJ3
18	CSF3
19	TNFRSF17
20	CD36
21	SOCS3
22	RPS4Y1
23	PIM2
24	FLG
25	FLG2
26	SEC11C
27	PTGS2
28	MMP3
29	RGS5
30	CCL18
31	RGS1
32	SFRP2
33	ZNF215
34	YOD1
35	CXCL1
36	AQP9
37	KDM5D
38	SLC2A3
39	ODAM
40	ALOX15B
41	HOOK1
42	SEL1L
43	CXCL10
44	SGMS2
45	IL10RA
46	TP53AIP1
47	XDH
48	COL1A1
49	CYP1B1
50	ICAM1
51	ENTPD1
52	STEAP4
53	CLDN20
54	RGS2
55	SPIN4
56	SORBS2
57	VPS41
58	BAMBI
59	PCDH18
60	BBS10
61	WDR74
62	CXCL6
63	GPR110
64	MALAT1
65	CXCR4
66	TXNDC11
67	PLA2G7
68	KCNJ2
69	EVI2B
70	IFI44L
71	SELL
72	NRCAM
73	NR4A2
74	GLCCI1
75	PANX1
76	BTG2
77	CRISP3
78	ABCA8
79	MYADM
80	PDK4
81	PLAUR
82	BPGM
83	LAMC2
84	ROR1
85	PRDM1
86	ICK
87	IFIT5
88	FMO1
89	ABCA12
90	STARD5
91	AVPI1
92	ARG1
93	VEGFA
94	SELK
95	CEACAM7
96	PSPH
97	HBB
98	ALDH1L2
99	BHLHE41
100	SLC16A9
101	BNC1
102	KRT19
103	FCGR2A
104	STK39
105	IL1A
106	CTGF
107	SLC7A5
108	EPCAM
109	ZNF273
110	DUSP5
111	MMP1
112	HLA-DQB1
113	GATM
114	F13A1
115	TNFRSF19
116	ADAM12
117	STXBP5
118	LYRM2
119	FOSB
120	IFIT1
121	PVRL4
122	MAN1A1
123	CDH19
124	TTC39A
125	NEFM
126	PIGW
127	EIF2AK2
128	CFB
129	STX11
130	GBP1
131	SLC39A8
132	DDX60L
133	ELTD1
134	CYP4X1
135	ACTA2
136	GBP2
137	DLX5
138	SOSTDC1
139	OAS1
140	TSPAN11
141	TNFRSF12A
142	ALYREF
143	NID1
144	SPAG17
145	LCE2B
146	PNLIPRP3
147	PLEKHM3
148	CHD9
149	PARM1
150	EPHA4
151	CNIH4
152	SFRP4
153	ANKRD22
154	EXOC5
155	CORIN
156	MS4A6A
157	MYO5B
158	ANXA3
159	ZFAND2A
160	BGN
161	PLP1
162	GLT8D2
163	SERPINE1
164	CRIP2
165	PALMD
166	TMEM117
167	JUND
168	CISD2
169	RNASE1
170	FAM103A1
171	CXCL12
172	STK17A
173	CORO1A
174	IGJ
175	MMP13
176	CALCRL
177	A2M
178	CORO1C
179	TGFBR3
180	GSTM1
181	TRIM5
182	FGFBP1
183	BMP2K
184	OLFML1
185	UFM1
186	SERPINB9
187	PMP22
188	FMO2
189	TRIM13
190	HIST2H2BE
191	C15orf41
192	PHACTR2
193	TCEA3
194	CDK8
195	CD177
196	CACHD1
197	SELP
198	ADH7
199	EEA1
200	HSPA4L

IL, interleukin; VEGFA, vascular endothelial growth factor A; ICAM1, intercellular adhesion molecule 1.

**Table II:** Pathway analysis based on the Kyoto Encyclopedia of Genes and Genomes.

Term	P-value	Count
Cytokine-cytokine receptor interaction	2.60E-06	16
Cell adhesion molecules	1.10E-02	7
Complement and coagulation cascades	1.60E-02	5
Intestinal immune network for Immunoglobulin A production	3.20E-02	4
Chemokine signaling pathway	4.80E-02	7
Drug metabolism	5.70E-02	4

**Table III:** Top 10 GO terms of the top 200 differentially expressed genes.

ID	Term	P-value	Count
GO:0006955	Immune response	6.60E-09	29
GO:0010033	Response to organic substance	1.10E-05	24
GO:0009611	Response to wounding	1.30E-05	20
GO:0016477	Cell migration	9.60E-05	13
GO:0006935	Chemotaxis	1.10E-04	10
GO:0042330	Taxis	1.10E-04	10
GO:0051674	Localization of cell	2.60E-04	13
GO:0048870	Cell motility	2.60E-04	13
GO:0032963	Collagen metabolic process	2.90E-04	5
GO:0042127	Regulation of cell proliferation	3.30E-04	22

**Table IV:** Genes with degrees >10 in the protein-protein interactions network.

Gene	Degree
IL8	28
IL1β	25
VEGFA	24
ICAM1	22
PTGS2, CXCL10	21
MMP1, CXCR4	17
CXCL12	16
COL1A1, CSF3	15
IL1A, SERPINE1	14
CD79A, SELL, PLAUR, MMP3	13
CXCL1, CTGF	12
SELP, SELE, MMP12, A2M	11

IL, interleukin; VEGFA, vascular endothelial growth factor A; ICAM1, intercellular adhesion molecule 1.
